# PLCD3 inhibits apoptosis and promotes proliferation, invasion and migration in gastric cancer

**DOI:** 10.1007/s12672-024-00881-w

**Published:** 2024-02-02

**Authors:** Yantao Yu, Shantanu Baral, Qiannan Sun, Jianyue Ding, Qi Zhang, Fanyu Zhao, Shuyang Gao, Qing Yao, Haoyue Yu, Bin Liu, Daorong Wang

**Affiliations:** 1 Dalian Medical University, Dalian, 116044 Liaoning China; 2 Yangzhou Clinical Medical College, Dalian Medical University, Yangzhou, 225001 Jiangsu China; 3 Clinical Medical College of Yangzhou University, Yangzhou, Jiangsu China; 4 Northern Jiangsu People’s Hospital, Yangzhou, 225001 Jiangsu China; 5 Medical Research Center of Northern Jiangsu People’s Hospital, Yangzhou, 225001, China; 6 General Surgery Institute of Yangzhou, Yangzhou University, Yangzhou, 225001 Jiangsu China; 7 Yangzhou Key Laboratory of Basic and Clinical Translation of Gastroenterology/Metabolic Diseases, Yangzhou, 225001 Jiangsu China

## Abstract

Gastric cancer (GC) is a heterogeneous disease whose development is accompanied by alterations in a variety of pathogenic genes. The phospholipase C Delta 3 enzyme is a member of the phospholipase C family, which controls substance transport between cells in the body. However, its role in gastric cancer has not been discovered. The purpose of this study was to investigate the expression and mechanism of action of PLCD3 in connection to gastric cancer. By Western blot analysis and immunohistochemistry, PLCD3 mRNA and protein expression levels were measured, with high PLCD3 expression suggesting poor prognosis. In N87 and HGC-27 cells, the silencing of PLCD3 using small interfering RNA effectively induced apoptosis and inhibited tumor cell proliferation, invasion, and migration. Conversely, overexpression of PLCD3 using overexpressed plasmids inhibited apoptosis in AGS and BGC-823 cells and promoted proliferation, migration, and invasion. In order to investigate the underlying mechanisms, we conducted further analysis of PLCD3, which indicates that this protein is closely related to the cell cycle and EMT. Additionally, we found that overexpression of PLCD3 inhibits apoptosis and promotes the development of GC cells through JAK2/STAT3 signaling. In conclusion, PLCD3 inhibits apoptosis and promotes proliferation, invasion, and migration, which indicated that PLCD3 might serve as a therapeutic target for gastric cancer.

## Introduction

Gastric cancer (GC) is one of the most common cancers worldwide, accounting for the fifth most common diagnosis. With a high mortality rate and often late diagnosis, it is the third leading cause of cancer-related death [1]. Men are more likely to suffer morbidity and mortality than women [2]. In the early stages of GC, common treatment options include endoscopic treatment and surgery [3]. It is generally considered to be a good postoperative prognosis, with a 5-year survival rate exceeding 90% [3]. However, in Europe, the recurrence rate for patients with advanced stages like IIIA/IIIB remains between 40 and 60% after surgical treatment, with a median survival time of 3 to 5 months without chemotherapy [4]. The treatment of advanced GC has always been challenging. Nevertheless, molecularly targeted therapy has played a key role in the development of advanced gastric cancer compared with conventional radiotherapy and chemotherapy. The most commonly targeted therapies modulate epidermal growth factor, angiogenesis, immune checkpoint inhibition, cell cycle, and apoptosis [5]. Targeted inhibitors can effectively regulate tumor cells’ biological behavior by regulating overexpressed molecules on tumor cells and signaling pathways closely related to tumorigenesis [6]. Therefore, to improve the therapeutic effect of gastric cancer, it is very imperative to better understand the mechanism of molecular targeted therapy. In addition, it is imperative to discover new molecular targets.

Phospholipase C Delta 3 (PLCD3) is a protein-coding gene located on chromosome 17aq21.31 [7]. It is a member of the phospholipase C family. This is made up of enzymes that hydrolyze phosphatidylinositol to create diacyl-glycerol (DAG) and inositol 1,4,5-triphosphate (IP3) [8]. The former mediates protein kinase C (PKC) activation [9], whereas the latter controls Ca^2+^ release from intracellular reserves [10, 11]. Two other members of the phospholipase C family are PLCD1 and PLCD4. In particular, PLCD1 has been identified as a tumour suppressor gene in 3p22, a region frequently mutated in esophageal cancer [12]. Additionally, PLCD1 inhibits pancreatic cancer cells' carcinogenic capacity [13]. Several reports have been published in recent years regarding the role of PLCD3 in cancer. As an example, PLCD3 promotes the proliferation, migration, and invasion of nasopharyngeal carcinoma cells [14]. The knockdown of PLCD3 in colon cancer impairs the development of microvillous structures and, as a result, promotes colon cancer development [15]. Despite this, no research has been undertaken on the role of PLCD3 in GC or the importance of molecular systems.

PLCD3 expression in gastric cancer and its predictive value were investigated in this study. To mute the expression of PLCD3 in GC cells, we employed small interfering RNA technology. Overexpressed plasmids were used to enhance the level of this gene in GC cells. In our study, PLCD3 knockdown promoted apoptosis and inhibited proliferation, invasion, and migration of GC cells. Conversely, overexpression of PLCD3 had the opposite effect. As a final step in our study, we investigated the combined effects of PLCD3 and its closely related pathways on the biological functions of gastric cancer.

## Materials and methods

### Bioinformatic analysis

NCBI-Gene Expression Omnibus (GEO) (https://www.ncbi.nlm.nih.gov/) is a public resource that includes microarray and high throughput sequencing data. A total of three microarray datasets have been selected and retrieved (GSE49051, GSE54129, and GSE112369). As a primary objective, we searched all datasets for gene expression patterns (overexpressed or underexpressed) specific to GC cells compared to their normal neighboring cells. To identify clinical characteristics linked to stomach cancer, the cancer genome atlas (TCGA) database was employed. Our analysis of Kyoto Encyclopedia of Genes and Genomes (KEGG) was conducted after integrating all the expression profiles using DAVID Bioinformatics and GSEA. For gene set enrichment analysis (GSEA), PLCD3 co-expressed genes were selected under the following conditions: enrichment analysis, KEGG pathway, rank criteria, *P*-value < 0.05, and minimum number of genes. We utilized the limma package in R software 4.3.2 to identify genes with distinct expression, using the cut-off criteria of |Fold change|> 0 (LogFc > 0) and *P*-Value < 0.05.

### Patients and GC specimens

Between July 2015 and May 2017, we collected 80 pairs of formalin-fixed and paraffin-embedded human GC specimens ranging in age from 43 to 77 years old. In addition, 16 pairs of freshly extracted GC tissues were taken from nine men and seven women aged 46 to 67. In this study Northern Jiangsu People's Hospital, School of Clinical Medicine, Yangzhou University (Yangzhou, China) provided the specimens. Notably, none of the patients had preoperative chemotherapy. Two distinguished pathologists verified stomach cancer diagnosis. The American Joint Committee on Cancer TNM staging approach for gastric cancer (AJCC-8 TNM) was utilized to assess the disease's stage. Northern Jiangsu People's Hospital, Yangzhou University School of Clinical Medicine and the Ethics Committee (Yangzhou, China) have all approved this project, with the permission number 2019 KY-022. Prior to the trial, all subjects provided written informed consent.

### Immunohistochemistry (IHC)

GC samples were fixed in paraffin and cut into 4 mm thick tissue slices. After that, the sections were fixed for one day in a 10% formalin solution before being placed on slides. The slides were baked in anoven at 60 °C for 2 h on the first day, and then allowed to cool to room temperature. In order to remove the wax, the tissue was treated with xylene and ethanol concentrations ranging from 100 to 50%. In the next step, sodium citrate (Cat#C1032, Solarbio, Beijing, China) was used for 30 min in order to repair the antigen. In order to block endogenous peroxidase activity, 3% H2O2 (PV-9000, OriGene, Jiangsu, China) was added. After treating the sample with goat serum (PV-9000, OriGene, Jiangsu, China) for 30 min, the primary antibody PLCD3 (1:50, PK83857, Abmart) was added dropwise. A secondary antibody (PV-9000, OriGene, Jiangsu, China) was added dropwise on the second day and incubated for 30 min at 37 °C. For nuclear staining, 1:20 DAB chromogenic solution (ZLI-9018, OriGene, Jiangsu, China) was used, followed by hematoxylin (Cat.C0107-100ml, Beyotime, Shanghai, China). In orderto photograph the blocking, an Olympus BX53 fluorescence microscope was used.

### Cell lines and cell culture

We obtained the normal human gastric cell line (GES-1) and five human GC cell lines (AGS, MKN45, BGC-823, N87, HGC-27) from the Cell Bank of the Chinese Academy of Sciences, Shanghai Institute of Cell Biology, Chinese Academy of Sciences, Typical Culture Repository. The cells were cultured in RPMI1640 medium (Cat. No. 31800, Solarbio, Beijing, China). The cells were cultivated at 37 °C in a 5% CO2 incubator (Thermo Scientific, USA) according to the manufacturer’s instructions. As a supplement to the medium, 10% fetal bovine serum (Lot.NM220304, Eallbio, Beijing, China), 100 units of penicillin and 100 units of streptomycin (60162ES76, Yeasen, Shanghai, China) were added. There was no sign of mycoplasma or any other fungi in any of the cell lines used in the experiment.

### Plasmid infection and transient transfection

GenePharma (Shanghai, China) constructed the small interfering PLCD3 (si-PLCD3) and negative control small interfering (siCtrl). The sequences are as follows: siCtrl (forward: 5′-UUCUCCGAACGUGUCACGUTT-3′, reverse: 5′-ACGUGACACGUUCGGAGAATT-3′); si1-PLCD3 (forward: 5′-GCAGCUCAUUCAGACCUAUTT-3′, reverse: 5′-AUAGGUCUGAAUGAGCUGCTT-3′); si2-PLCD3 (forward: 5′-GCCCACUACUUCAUCUCUUTT-3′, reverse: 5′-AAGAGAUGAAGUAGUGGGCTT-3′). A pcDNA-PLCD3 construct was cloned into the BamH1/EcoRI restriction digest site of the pcDNA3.1 plasmid by GenePharma. The primer sequence for PLCD3 with the BamH 1/EcoRI enzyme site is as follows: oe-PLCD3 (forward: 5′-GCTTGGTACCGAGCTCGGATCCGCCACCATGCTGTGCGGCC-3′, reverse: 5′-TGCTGGATATCTGCAGAATTCTCAGGAGCGCTGGATGCGGATTTGGATGA-3′). 2 × 10^5^ GC cells were inoculated into six-well plates and grown overnight. The plasmid was then infected and transiently transfected with lipofectamine 2000 (Lot.2357812, Invitrogen, USA) the next day. Transfected cells were incubated at 37 °C in a 5% CO2 incubator for 48 h.

### Real-time quantitative PCR(RT-qPCR) assays

The total RNA was extracted from the cell lines using Trizol reagent (R701-01-AA, Vazyme, Nanjing, China). We transcribed the extracted RNA into cDNA using a reverse transcription kit (017E2282IA, Vazyme, Nanjing, China) and performed RT-qPCR using a SYBR Green PCR Kit kit (Cat:11203ES08, Yeasen, Shanghai, China) as described in a previous study [16]. Using StepOne Software v2.3, Thermo Fisher Scientific, USA, the amplification procedure was a two-step process involving 95 °C for 5 min, 95 °C for 10 s, and 60 °C for 30 s, with a cycle count of 40. Primers were provided by Sangon Biotech, Shanghai, China, and their sequences were as follows: PLCD3-forward (5′-CATTCGGGAGGCAGGGAAC-3′), PLCD3-reverse (5′-TTCCACATCTCCTGGGGACT-3′). GAPDH primer sequences are as follows: GAPDH forward (5′-TGACATCAAGAAGGTGGTGAAGCAG-3′) and GAPDH reverse (5′-GTGTCGCTGTTGAAGTCAGAGGAG-3′). We used the 2^−ΔCt^ method using GAPDH as an internal reference to determine the relative expression of the targets. RedSafe nucleic acid staining solution was used to visualize PCRproducts after electrophoresis on 1% agarose gels.

### Western blot

Cells were lysed with RIPA lysis solution (Cat# R0010, Solarbio, Beijing, China) at 4 °C. A lysate of scraped cells was collected, centrifuged at 14,000 rpm for 15 min at 4 °C, and the supernatant was removed. The absorbance values of the cells were measured at 570 nm using the BCA kit (No.927E2220KA, Vazyme, Nanjing, China). The samples were boiled for 10 min after being mixed with 5X Loading buffer. Afterwards, they were electrophoresed on an SDS-PAGE gel and electrotransferred to a PVDF membrane (Lot: 0000161247, Immobilon-P, Merck Millipore, Ireland). Closure with 5% skimmed milk at room temperature for 90 min, followed by overnight shaking at 4°C with the primary antibody. The following antibodies were included in the study: PLCD3 (1:1000, Abmart), GAPDH Mouse mAb (1:10,000, abclonal), BAX (1:1000, ZENBIO), P53 (1:1000, ZENBIO), BCL2 (1:1000, ZENBIO), MDM2 (1:1000, Cell Signaling Technology), CDK2 (1:1000, ZENBIO), CDK6 (1:1000, ZENBIO), MTOR (1:1000, ZENBIO), P-MTOR (1:1000, ZENBIO), MMP2 (1:1000, Cell Signaling Technology), MMP9 (1:1000, Cell Signaling Technology), JAK2(1:1000, Cell Signaling Technology), P-JAK2 (1:1000, Cell Signaling Technology), STAT3 (1:1000, Cell Signaling Technology), P-STAT3 (1:2000, Cell Signaling Technology), N-Cadherin (1:1000, Affinity), E-Cadherin (1:1000, Affinity). The following day, secondary antibodies Goat anti-Rabbit IgG (1:8000, ZENBIO) and Goat anti-Mouse IgG (1:8000, ZENBIO) were incubated at room temperature for 1h, followed by development with ECL Immunoblot Detection Reagent (Cat.#KF8003, Affinity, USA).

### Cell Counting Kit‑8 (CCK‑8) assay

In each well of a 96-well plate (Lot:111622BL01, NEST, Wuxi, China), 1000 cells were inoculated and incubated overnight at 37 °C. The CCK-8 reagent (Lot#255,919, MedChemExpress, USA) was added at 24 h, 48 h, 72 h, and 96 h following the addition of the CCK-8 reagent. After 2 h of incubation, the absorbance value of each well was measured using a microplate reader (Thermo Scientific, USA).

### Colony formation assay

In six-well plates, 600 cells were spread per well after small interferences and plasmid infection. The cells were washed three times with PBS after two weeks of culture, then fixed in 4% paraformaldehyde (Lot22360481, Biosharp, Beijing, China), and stained with 5% crystalline violet solution (Cat# G1075, Solarbio, Beijing, China). Imagej (National Institutes of Health, Bethesda, USA) software was used to image and count the cell colonies as described in previous studies.

### Wound healing assay

The cells were inoculated into 6-well plates (Corning, USA) and cultured for one day in serum-free RPMI-1640 medium. After the cell density reaches 80–90%, the monolayer is scraped vertically downwards using a yellow tip with a range of 200 mL and the residual cells are washed with PBS. After that, the medium was changed to 2% FBS. At 0 h, 24 h, and 48 h, the cells were observed under a microscope (OLYMPUS CKX53, France) and the migration phenomenon was recorded.

### Transwell migration and invasion assays

The migration experiments were conducted using small chambers (353,095, Corning, USA) in 24-well plates (Lot:061722BH01, NEST, Wuxi, China). In the small chamber, the upper chamber is filled with 200 ml of RPMI-1640 cell suspension without FBS (number of cells: 1 × 10^4^) and the lower chamber is filled with 500 ml of RPMI-1640 medium containing 10% FBS. For invasion experiments, 10% artificial substrate gel (cat. no.356234, BD Biosciences, USA) was spread in the upper chamber of the small chamber the night before the experiment, 200 L of FBS-free medium was added to the upper chamber and 500 L of complete medium was added to the lower chamber. They were fixed with 4% paraformaldehyde for 24 h before dyeing with 5% crystalline violet solution. A light microscope was used to capture the images, and five randomly selected fields of view were analyzed statistically.

### Immunofluorescence staining

N87, HGC-27, AGS, and BGC-823 cell lines were transfected the night before and plated in confocal culture dishes (Cat. No.: 801001, NEST, Wuxi, China). The cells were rinsed three times in PBS the next day before being fixed in 4% paraformaldehyde for 15 min at room temperature. They were then permeabilized with 0.1% Triton X 100 for 3 min. The cells were blocked with normal goat serum (Cat.No. SLO38, Solarbio, Beijing, China) for 30 min at room temperature and then incubated with diluted primary antibody PLCD3 (1:100, Bioss, Beijing, China) overnight at 4 °C. A fluorescent secondary antibody, Goat Anti-Rabbit IgG (H + L) Fluor594-conjugated (1:100, Affinity, USA), was added dropwise in a dark room, incubated for 1 h, and stained with DAPI (C1005, Beyotime, Shanghai, China) for 10 min the following day. The images were finally acquired by observation with a laser confocal microscope (Carl Zeiss LSM710, Germany) after four washes with PBS.

### Statistical analysis

Statistical analysis was performed using GraphPad Prism 9 (San Diego, CA, USA). The Student’s *t*-test was used to compare differences between the two groups. ANOVA was used for groups of three or more. Kaplan–Meier survival curves and the log-rank sum test were used to analyze the survival curves. According to the following criteria, statistical significance was determined: *P < 0.05, **P < 0.01, ***P < 0.001, and ****P < 0.0001.

## Results

### PLCD3 is significantly overexpressed in the GC

**Fig. 1:**
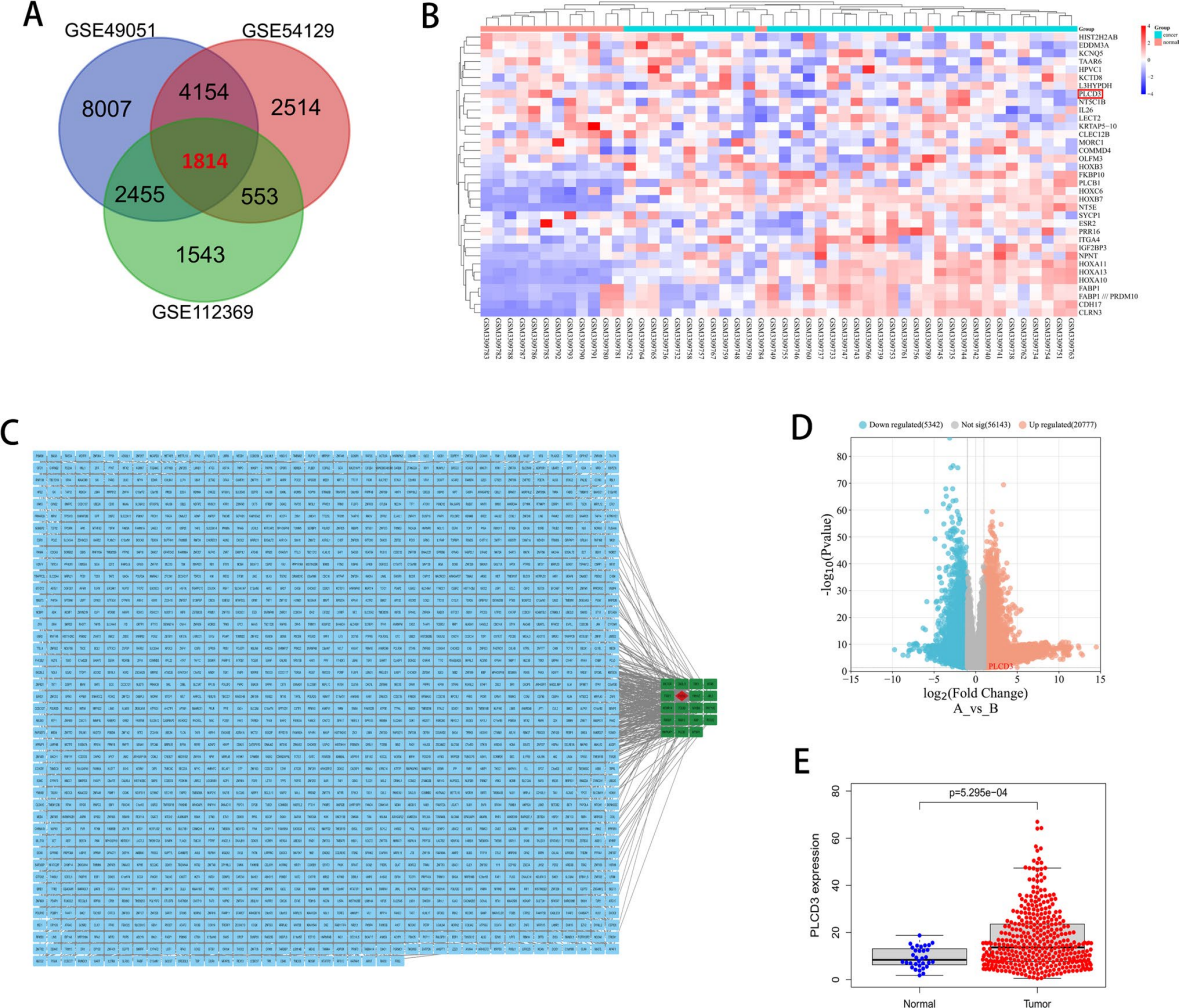
PLCD3 was up regulated in gastric cancer (GC) and was associated with pathological grades based on online databases. **A** A total of 1814 genes were differentially expressed (fold change > 0 and *P* < 0.05) between tumor tissues and the adjacent normal tissues from two GEO profiles (GSE49051, GSE54129, and GSE112369). **B** The Heat map showed the relative expression of 40 genes according to the *p*-value. **C** Interaction network of differentially expressed mRNAs. **D** The Volcano map showed all differentially expressed genes. **E** PLCD3 expression was compared between tumor tissues and the adjacent tissues from TCGA database (*P* = 5.29e−04)

Based on two GEO profiles, we identified 1814 common genes using the criterion of |log2 fold change|> 0 and *P* < 0.05 (Fig. 1A). We created heat maps and volcano maps to display these genes between gastric tumor samples and associated normal tissues (Fig. 1B, D). The PPI interaction shown in (Fig. 1C) contains 1767 nodes and 17,551 edges. MCODE was used to identify 47 clusters. The major cluster with the highest MCODE score, 6.423, is highlighted in (Fig. 1C). There are 19 nodes and 34 edges in the major cluster. The 19 nodes represent the DEGs that have been up regulated in GC (Table 1). In addition, the expression of PLCD3 in tumor tissues was higher when compared to the expression in normal tissues in the TCGA database (Fig. 1E). These findings suggest that PLCD3 may play a key role in gastric cancer genesis and progression.

**Table 1:** The differentially expressed upregulated major cluster genes in gastric cancer

Genes	Fold change (tumor/normal)	*P*-Value
RICTOR	1.3242939	0.00000416
DIABLO	1.2851931	0.000000921
TBK1	0.9613753	0.0000154
MTM1	1.3764494	0.0000241
TRAF3	2.0950505	0.0196
PLCD3	1.0348227	0.0261
YWHAZ	1.0683431	0.00000817
ARL3	0.3906021	0.00962
MTMR14	1.9550596	0.000183
PDE6D	0.6043461	0.0000175
NFKBIB	1.9721522	0.0000133
RAB3IP	2.8438078	7.06E−08
RAB10	0.5553915	0.0000509
XIAP	1.4555047	0.00000166
ROCK2	1.3112292	0.000000734
MAPKAP1	1.6317335	0.000000855
PLCB3	2.9588767	0.000273
MTMR1	0.8808859	0.0000106

### PLCD3 is upregulated in GC and upregulation is associated with poor prognosis in GC patients

**Fig. 2:**
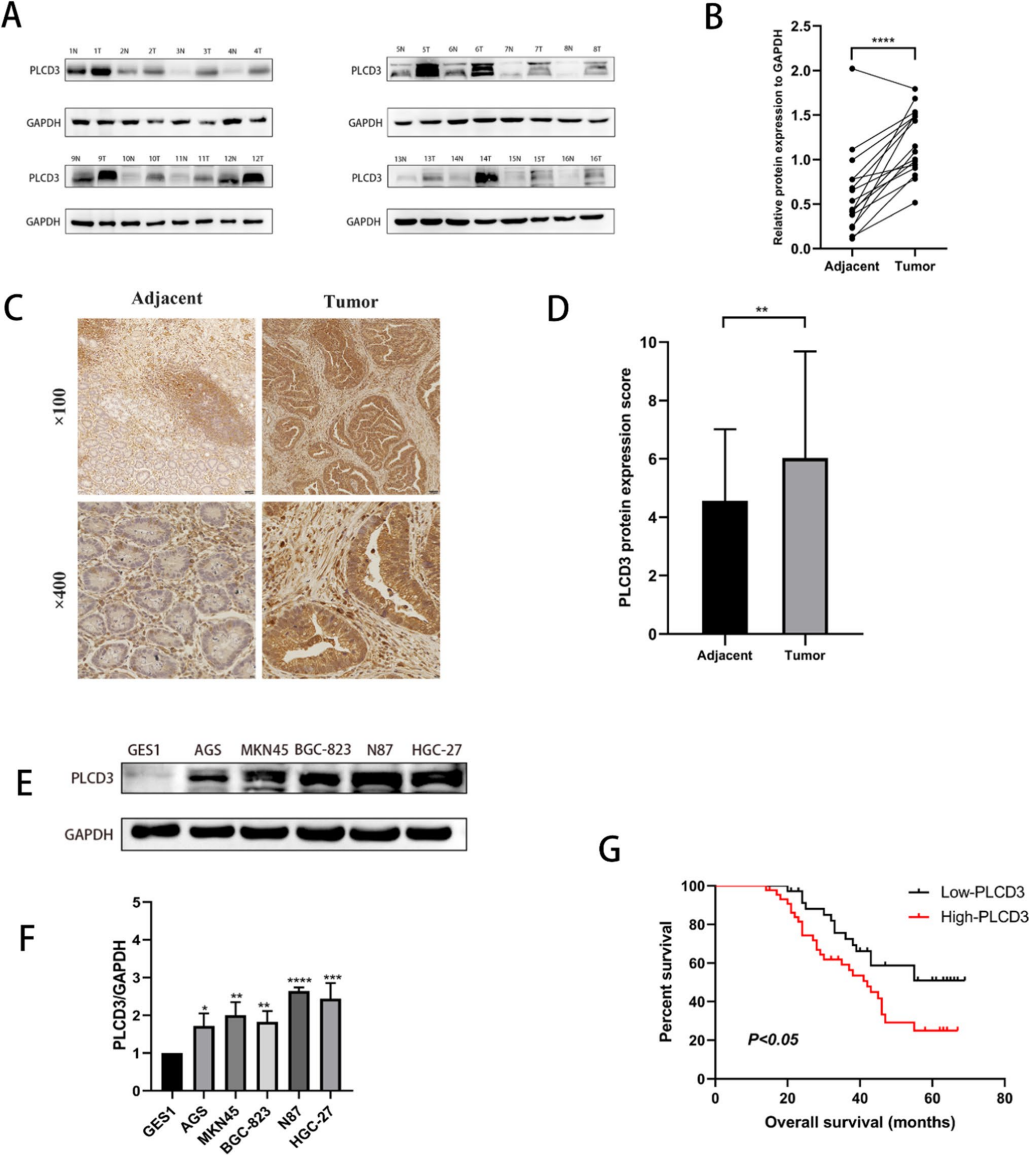
PLCD3 expression is upregulated in GC cells and tissues. **A** Western blot analysis results of PLCD3 in 16 pairs of adjacent tissue and GC tissue. **B** PLCD3 expression in 16 paired GC and adjacent tissue samples was determined by western blotting (****P < 0.0001). **C** Immunohistochemical analysis of PLCD3 in paired noncancerous tissues and GC tissues of patients (n = 80). **D** Qualification of PLCD3 staining in TMAs in **F**. The graph depicts the total score, the multiplication product of staining intensity and the percentage of stained cells (**P < 0.01). **E**, **F** The protein expression levels of PLCD3 in GC cell lines (AGS, MKN45, BGC-823, N87, HGC-27) and normal gastric cell line GES-1. GAPDH served as a loading control (*P < 0.05, **P < 0.01, ***P < 0.001, and ****P < 0.0001). **G** Survival analysis of gastric cancer patients with different expression levels of PLCD3. N: Normal; T: Tumor

A western blot analysis was conducted on 16 pairs of fresh specimens from patients with GC to confirm the high expression of PLCD3 reported in the TCGA database. PLCD3 expression was significantly higher in GC tissues than in paracancerous tissues (*P* = 0.0001; Fig. 2A, B). Furthermore, immunohistochemical examination of 80 GC specimens revealed that PLCD3 was considerably overexpressed in GC tissue vs paracancerous tissue (Fig. 2C, D). As well as validating at the tissue level, we conducted studies at the cellular level. Western blot analysis of the cell lines (Fig. 2E, F) revealed that PLCD3 was highly expressed in five other GC cell lines (AGS, MKN45, BGC-823, N87, HGC-27) in comparison with the normal cell line GES1. Furthermore, the relationship between PLCD3 expression and clinicopathological features was studied. The results showed that PLCD3 expression was associated with age (*P* = 0.007), tumor size (*P* = 0.008), Laure type (*P* = 0.010), and TNM stage (*P* = 0.025). PLCD3 expression, however, was not associated with gender (*P* = 0.408), depth of invasion (*P* = 0.964), Lymphonodus metastasis (*P* = 0.665), distant metastasis (*P* = 0.084), degree of differentiation (*P* = 0.187), hsitological grade (*P* = 0.506), venous invasion (*P* = 0.463) or nerve invasion (*P* = 0.284) (Table 2).

**Table 2:** Associations between PLCD3 expression and clinicopathological features of 80 patients with gastric cancer

Histopathological parameters	Total number (80)	Expression level of PLCD3		*P*-value
		Low	High	
Age, years				0.007
< 65	40	25	15	
≥ 65	40	13	27	
Gender				0.408
Male	51	26	25	
Female	29	12	17	
Tumor size				0.008
< 6	38	24	14	
≥ 6	42	14	28	
Lauren type				0.010
Intestinal type	24	17	7	
Diffuse type	56	22	34	
Depth of invasion				0.964
T1, T2	38	17	21	
T3, T4	42	19	23	
Lymphonodus metastasis				0.665
N0, N1	23	8	15	
N2, N3	57	17	40	
Distant metastasis				0.084
Negative	58	31	27	
Positive	22	7	15	
TNM stage				0.025
I–II	40	24	16	
III–IV	40	14	26	
Degree of differentiation				0.187
Highly	29	14	15	
Moderately and poorly	51	17	34	
Histological grade				0.506
I	8	5	3	
II	38	19	19	
III	34	14	20	
Venous invasion				0.463
No	45	23	22	
Yes	35	15	20	
Nerve invasion				0.284
No	35	19	16	
Yes	45	19	26	

**Fig. 3:**
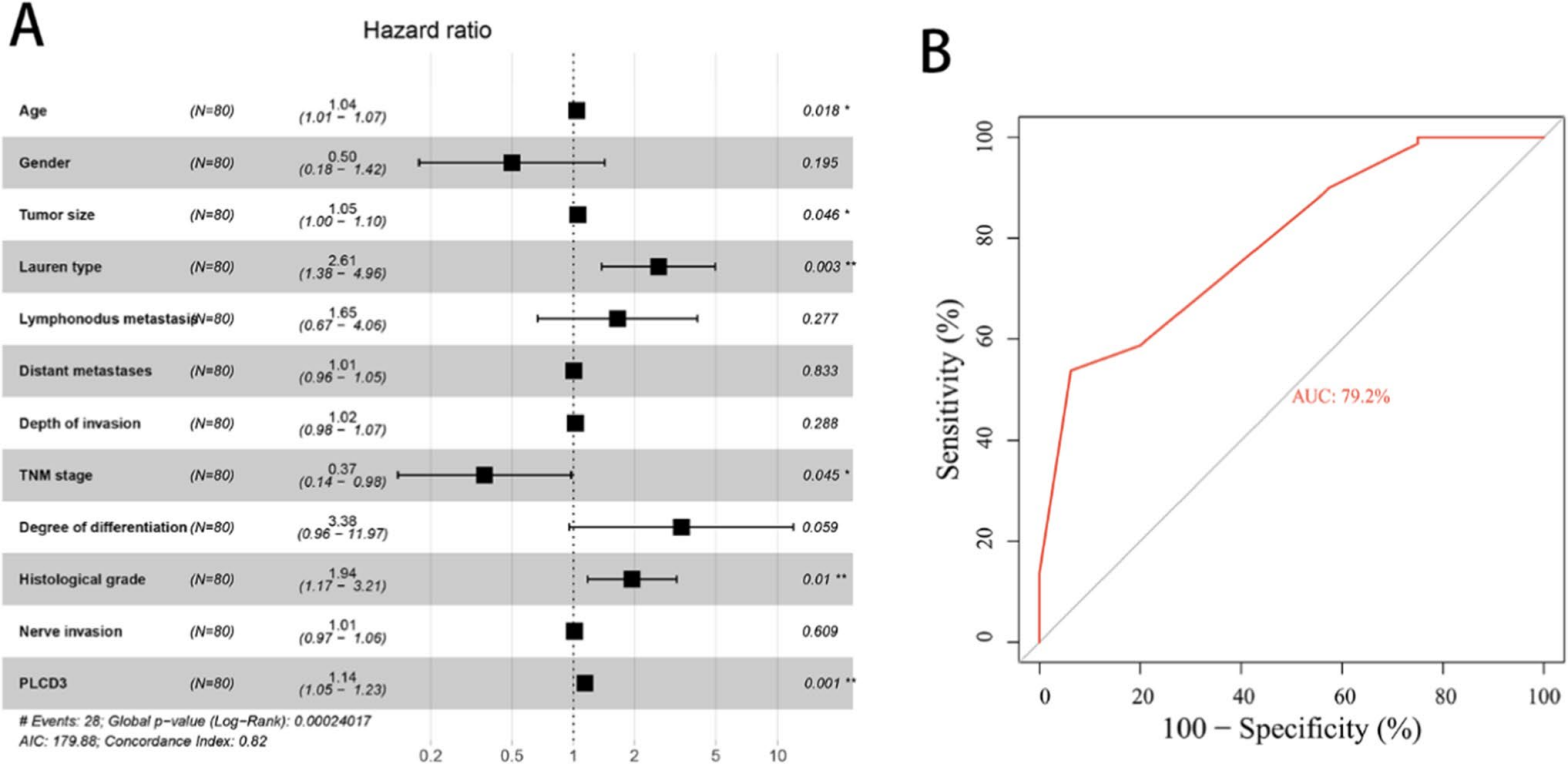
Clinical significance of the expression of PLCD3. **A** Forest plot of multivariate analysis in the validated cohorts. **B** The ROC curve for expression of PLCD3 illustrated excellent specificity and sensitivity which the AUC is 79.2%

The number of positive cells and the intensity of staining are used to assess PLCD3 staining. The staining intensity is measured as follows: Colorless counts as 0, yellow equals 1, brown equals 2, and dark brown is 3. The scores are based on the percentage of positive cells and are as follows: 0 (0–5%), 1 (5–25%), 2 (25–50%), 3 (50–75%) and 4 (> 75%). In order to calculate the final score, the intensity of staining and the percentage of positive cells are multiplied together. The results were scored independently by two expert pathologists. As per previous studies, we consider scores of ≥ 5 as positive expressions and scores of ≤ 4 as negative expressions [17]. In order to investigate the relationship between the expression of PLCD3 and the prognosis of GC, 80 patients with GC were evaluated for the association between PLCD3 expression and overall survival (OS). Based on Kaplan–Meier analysis, GC patients with high PLCD3 expression had a significantly lower 5-year survival rate than those with low PLCD3 expression (*P* < 0.05; Fig. 2G). Using univariate regression analysis, we found the following factors to influence OS (*P* > 0.05): age, tumor size, Lauren type, TNM stage (stages I and II vs. stages III and IV), histological grade (stages I vs. II vs. III), and PLCD3 expression (low vs. high) (*P* > 0.05). The following conclusions were obtained from multivariate regression analysis for the independent risk variables for GC development (*P* < 0.05, Table 3, Fig. 3A): The multivariate regression analysis revealed that age (*P* = 0.0018), tumor size (*P* = 0.046), Lauren type (*P* = 0.003), TNM stage (*P* = 0.0045), and histological grade (*P* = 0.01) and increased expression of PLCD3 (*P* = 0.001). According to these findings, PLCD3 levels are increased in GC patients, and patients with high PLCD3 levels have a poor prognosis. Following this, we used ROC analysis to determine the clinical diagnostic value of PLCD3 expression. The results (Fig. 3B) for expression of PLCD3 illustrated excellent specificity and sensitivity which the AUC is 79.2%.

**Table 3:** Prognostic factors on overall survival were analyzed by univariate Cox’s proportional hazards models in 80 patients with gastric cancer

	Univariate analysis	*P*-value
	HR (95%CI)	
Age	1.035(1.007–1.063)	0.012
Gender	0.689(0.312–1.523)	0.357
Tumor size	1.046(1.006–1.087)	0.024
Lauren type	1.237(1.004–1.525)	0.046
Lymphonodus metastasis	0.874(0.404–1.893)	0.733
Distant metastases	0.994(0.962–1.026)	0.694
Depth of invasion	1.021(0.986–1.056)	0.244
TNM stage	1.459(1.046–2.035)	0.026
Degree of differentiation	2.717(0.928–7.866)	0.065
Histological grade	1.035(1.007–1.063)	0.012
Nerve invasion	0.993(0.966–1.022)	0.645
PLCD3	1.673(1.105–2.533)	0.015

HR, hazard ratio; CI, confidence interval

### Transfection of small interfering RNAs and infection with overexpressed plasmid effectively regulate the expression of PLCD3

**Fig. 4:**
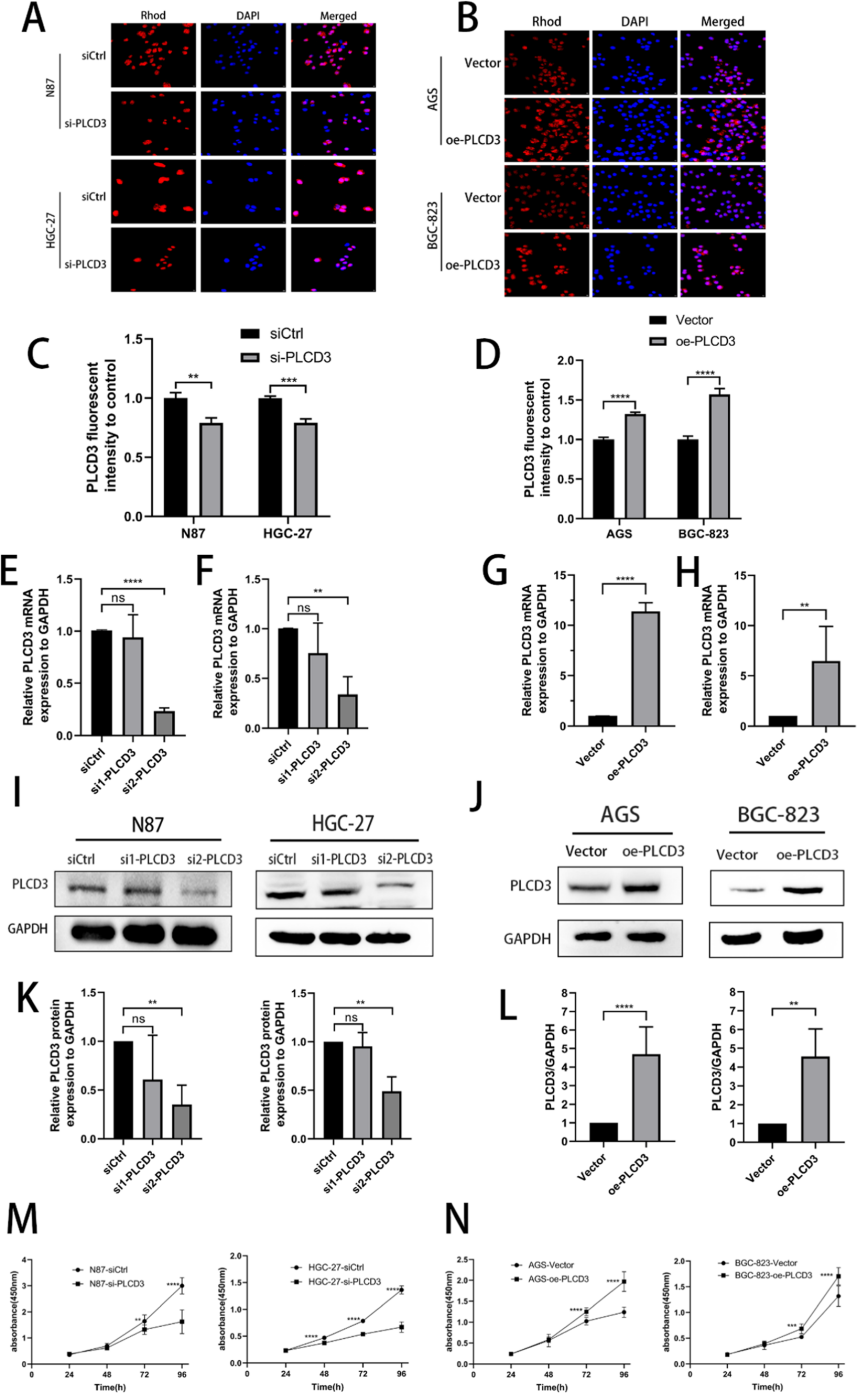
Knockdown of PLCD3 inhibited the proliferative capacity of gastric cancer cells and overexpression of PLCD3 increased the proliferative capacity of gastric cancer cells. **A** Immunofluorescence results of N87 cell line and HGC-27 cell line after knockdown of PLCD3. **B** Immunofluorescence results of AGS cell line and BGC-823 cell line after overexpression of PLCD3. **C**, **D** Fluorescence intensity of PLCD3 for each cell line compared to the control (**P < 0.01, ***P < 0.001, and ****P < 0.0001). **E**, **F** Knockdown efficiency of PLCD3 in the si-PLCD3 group in N87 and HGC-27 cells as measured by reverse transcription quantitative PCR (**P < 0.01, ****P < 0.0001, ns means not statistically significant). **G**, **H** Overexpression efficiency of PLCD3 in the oe-PLCD3 group in AGS and BGC-823 cells as measured by reverse transcription quantitative PCR (**P < 0.01, ****P < 0.0001, ns means not statistically significant). **I**, **K** Silencing of PLCD3 protein expression levels in N87 cell line and HGC-27 cell line (**P < 0.01, ns means not statistically significant). **J**, **L** Protein expression levels of overexpressed PLCD3 in AGS cell lines and BGC-823 cell lines (**P < 0.01, ****P < 0.0001, ns means not statistically significant). **M**, **N** The proliferation of N87 cells, HGC-27 cells after PLCD3 knockdown and AGS cells, BGC-823 cells after PLCD3 overexpression by Cell CountingKit‑8 assay. (**P < 0.01, ***P < 0.001****P < 0.0001). si: Small interfering; oe: overexpression

Using PLCD3 expression levels for each of the GC cell lines, we identified N87 and HGC-27 as potential targets for knockdown. Out of two small interferences, we selected the longer sequence (si2-PLCD3). Furthermore, we selected AGS and BGC-823 as our overexpression groups. First, we validated the two groups of cells using immunofluorescence techniques. The fluorescence intensity of PLCD3 was greater in the blank group than in the knockdown group in HGC-27 and N87 cells (Fig. 4A, C). Conversely, in AGS and BGC-823 cells, the fluorescence intensity of PLCD3 was observed to be lower in the blank group as compared to the overexpression group (Fig. 4B, D). Our reagents and interference methods were effective and accurate. Furthermore, in both N87 and HGC-27 cells, the expression of PLCD3 was lower in the si-PLCD3 group compared to the control group. The expression of PLCD3 was higher in the oe-PLCD3 group compared with the control group in AGS and BGC-823 cells. On transfected and infected cells, RT-qPCR was performed. In N87 and HGC-27, si-RNA1 was ineffective in reducingPLCD3 transcript levels, while si-RNA2 knockdown was statistically significant (Fig. 4E, F). The PLCD3 overexpression plasmid was effective in both AGS and BGC-823 cell lines (Fig. 4G, H). Finally, Western blot analysis of the cells under investigation revealed that protein translation was consistent with transcriptional levels, as expected (Fig. 4I–L).

### PLCD3 regulates proliferation, migration and invasion of gastric cancer

**Fig. 5:**
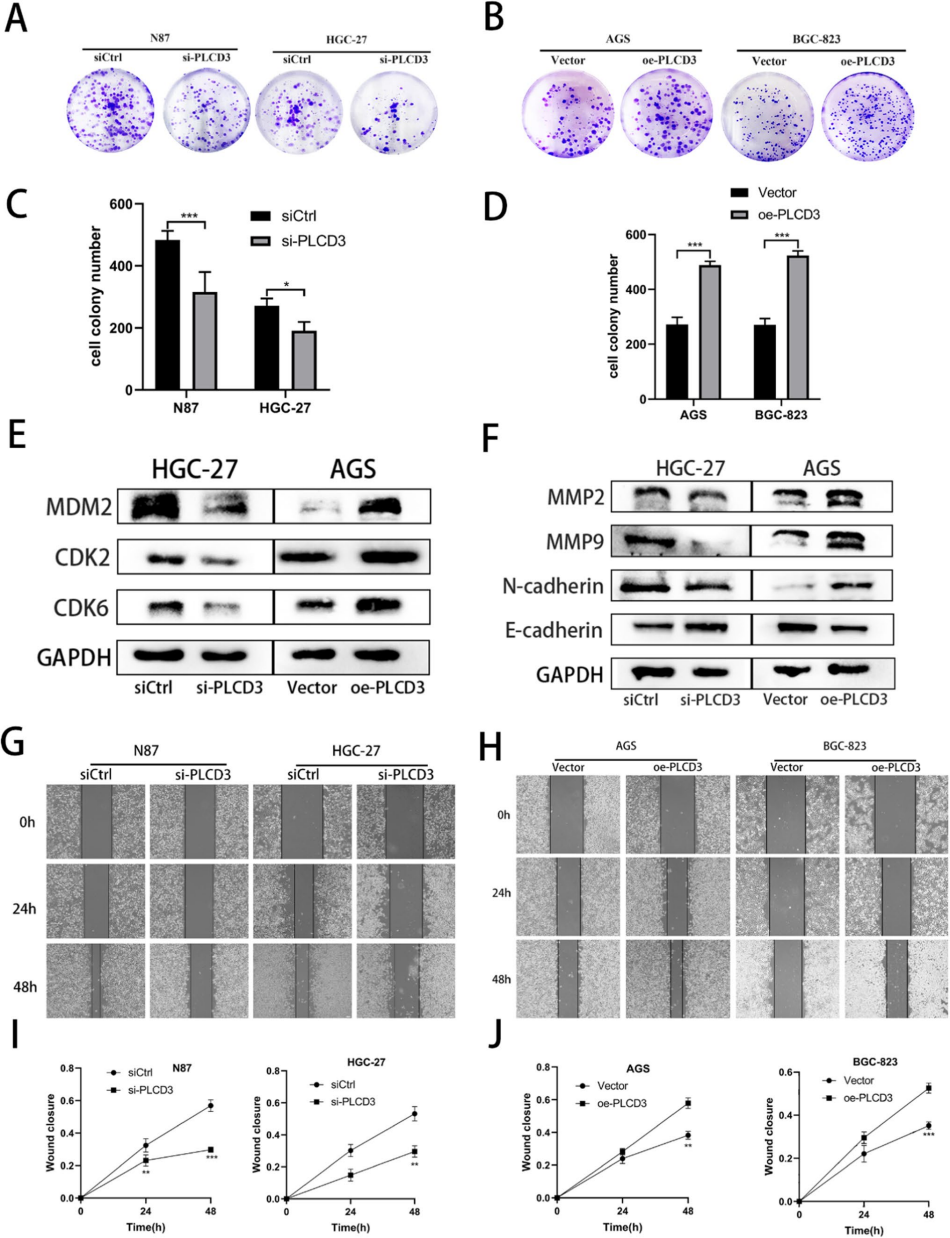
Knockdown of PLCD3 inhibited GC cell proliferation, migration, and overexpression of PLCD3 promotes GC cell proliferation migration. **A**, **C** Detection of the proliferation of N87 and HGC‑27 cells after PLCD3 knockdown by colony formation assays (*P < 0.05,***P < 0.001). **B**, **D** Detection of the proliferation of AGS and BGC-823 cells after PLCD3 overexpression by colony formation assays (***P < 0.001). **E** Western blot assays showed that knockdown of PLCD3 in HGC-27 cell line inhibited the expression of MDM2, CDK2, CDK6 and overexpression of PLCD3 in AGS cell line promoted the expression of MDM2, CDK2, CDK6. GAPDH was used as the loading control. **F** Western blot assays showed that silence of PLCD3 in HGC-27 cell line inhibited the expression of MMP2, MMP9, N-cadherin, E-cadherin and overexpression of PLCD3 in AGS cell line promoted the expression of MMP2, MMP9, N-cadherin, E-cadherin. GAPDH served as a loading control. **G**, **I** Detection of the migration of N87 and HGC‑27 cells after PLCD3 knockdown by wound healing assay (**P < 0.01, ***P < 0.001). **H**, **J** Detection of the migration of AGS and BGC-823 cells after PLCD3 overexpression by wound healing assay (**P < 0.01, ***P < 0.001)

**Fig. 6:**
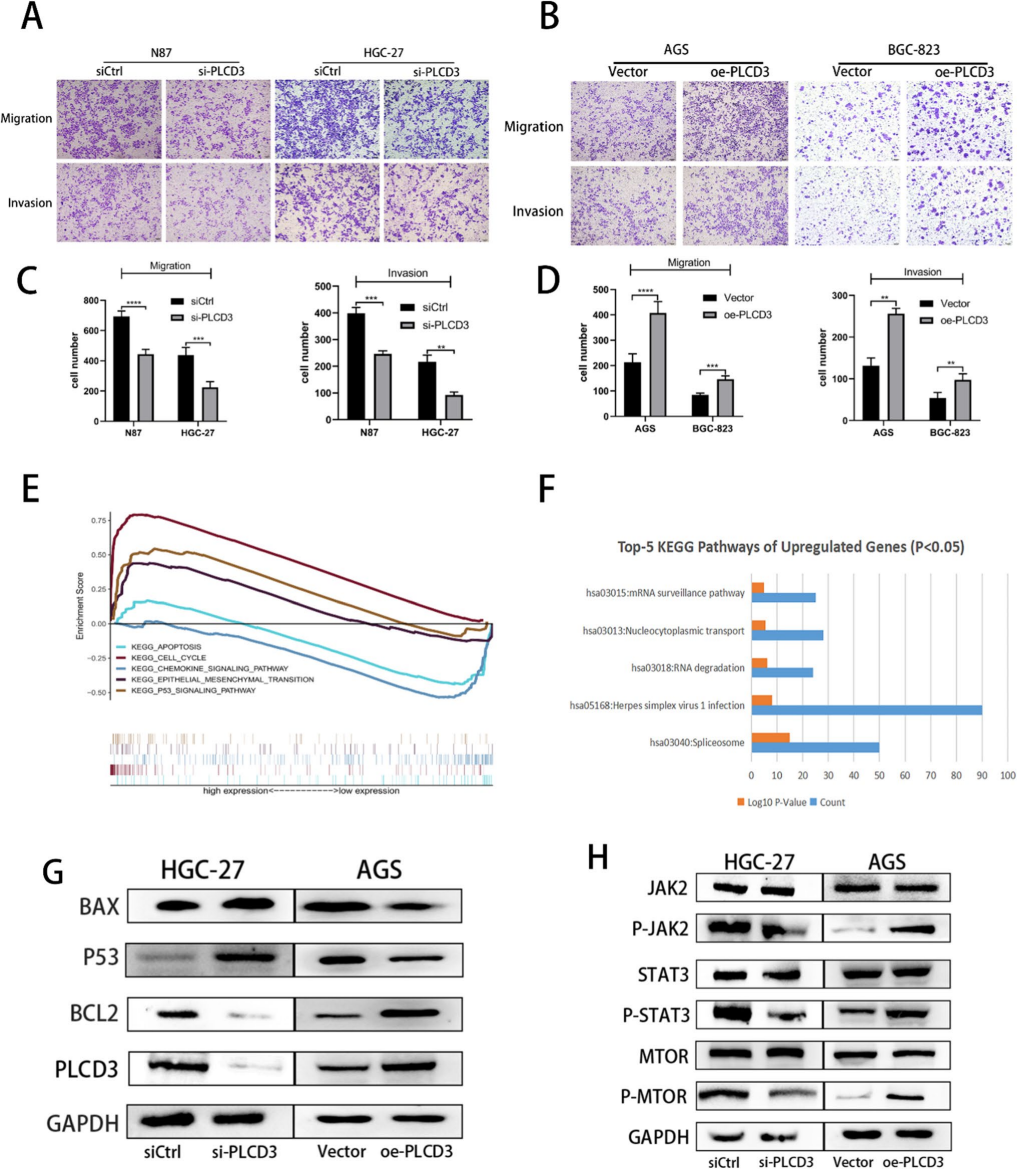
Knockdown of PLCD3 promotes apoptosis and inhibits cell migration and invasion in GC cells and overexpression of PLCD3 inhibits apoptosis and promotes cell migration and invasion in GC cells, and PLCD3 regulates the JAK2/STAT3 pathway. **A**, **C** N87 and HGC-27 cells after PLCD3 knockdown and **B**, **D** AGS and BGC-823 cells after PLCD3 overexpression (**P < 0.01, ***P < 0.001, ****P < 0.0001). **E** The top-5 most significantly enriched GSEA-KEGG terms. **F** The top 5 significantly enriched KEGG pathways. **G** The protein expression of the markers of the apoptosis pathway (BAX, P53 and BCL2) determined by western blot analysis. **H** Western blot assays showed that expression of proteins related to the JAK2/STAT3 pathway. GAPDH was used as the loading control

Based on our previous explorations, we chose si2-RNA-transfered cells for our cell function experiments. The CCK8 assay confirmed that PLCD3 influences cell growth following transient transfection. When knockdown PLCD3 cells were compared to control cells at 72 and 96 h in N87 cells, the findings (Fig. 4M) revealed a substantial reduction in proliferation ability. When compared to the blank control, PLCD3 knockdown significantly reduced HGC-27 proliferation at 48 h. AGS and BGC-823 cells infected with plasmid showed a higher proliferation rate than cells infected with an empty plasmid (Fig. 4N). In order to demonstrate the cells’ proliferative capacity, we performed the plate clone formation assay (Fig. 5A, B). It was found that in N87 and HGC-27 cells inhibiting PLCD3, the proliferation was inhibited, whereas in AGS and BGC-823 cells overexpressing PLCD3, the proliferation was enhanced (Fig. 5C, D). There is a strong correlation between the development of gastric cancer and its ability to migrate and invade. PLCD3 has a crucial function in controlling gastric cancer migratory ability, as revealed by wound healing and transwell assays. In both N87 and HGC-27 cells, the si-PLCD3 group exhibited a reduced cell migration capacity at 24h and 48h compared to the control group (Fig. 5G, I). On the other hand, the oe-PLCD3 group demonstrated a higher cell migration capacity at 24 h and 48 h compared with the control group in both AGS and BGC-823 cells (Fig. 5H, J). In addition, the Matrigel invasion assay demonstrated that blocking PLCD3 significantly reduced GC cell invasion (Fig. 6A, C), whereas overexpression of PLCD3 significantly increased it (Fig. 6B, D).

### KEGG pathway enrichment analysis for PLCD3 in GC

The KEGG pathway enrichment analysis of DEGs was carried out using the GSEA approach (Fig. 6E). GSEA was significantly enriched in pathways involved in APOPTOSIS, CELL CYCLE, CHEMOKINE SIGNALING, EPITHELIAL MESENCHYMAL TRANSITION, and P53 SIGNALING. Using Enrichr, we conducted pathway enrichment analysis of the hub genes. By using Enrichr, 17 enriched KEGG pathways were identified, of which 5 showed *P* < 0.05 (Fig. 6F). In the first enriched pathway, surveillance pathway, five genes (HBS1L, DAZAP1, RBM8A, CSTF3, and CSTF2T) overlap, all of which have been previously implicated in cancer progression. Among the main cluster of genes, the Herpes simplex virus 1 infection pathway contains the most genes.

### PLCD3 affects the cycle of GC cells

Since PLCD3 has the ability to regulate cell proliferation, we wanted to investigate whether it affects the cell cycle. In the G1-S and G2-M phases of the cell cycle, the cell cycle is tightly regulated. Figure 5E shows that in HGC-27 cells, the expression of CDK2 and CDK6 was reduced in the si-PLCD3 group as compared to the siCtrl group. Conversely, in AGS cells, CDK2 and CDK6 expression was increased in the oe-PLCD3 group compared to the Vector group. The results of this study suggest that PLCD3 is involved in the G1 and S phases of the cell cycle. Furthermore, PLCD3 has been shown to alter the G2/M phase of the cell cycle in a way similar to MDM2. As a result, by influencing the expression of these proteins, PLCD3 plays a significant role in regulating the cell cycle.

### PLCD3 is closely associated with epithelial-mesenchymal transition (EMT)

Knockdown of PLCD3 inhibited gastric cancer migration and invasion in both wound healing and transforaminal migration and invasion assays. To explore the potential mechanism, we conducted Western Blot experiments. The loss of cell-to-cell adhesion and polarity, the down-regulation of epithelial markers, and the acquisition of mesenchymal markers and phenotypes describe an epithelial-to-mesenchymal transition [18]. As a consequence, Western blot analysis was carried out to confirm the relationship between PLCD3 and the epithelial-mesenchymal transition (EMT) in GC. PLCD3 knockdown lowered the levels of MMP2, MMP9, and N-cadherin while increasing E-cadherin (Fig. 5F). On the other hand, overexpression of PLCD3 had the opposite effect. According to these findings, PLCD3 facilitates the migration and invasion of GC cells via epithelial mesenchymal transition (EMT).

### PLCD3 is involved in the apoptotic process of GC cells

Apoptosis is a kind of controlled cell death triggered by developmental stimuli or physiological stress [19]. In order to investigate the relationship between PLCD3 and apoptosis, we conducted western blot analysis on HGC-27 cells in the si-PLCD3 group and AGS cells in the oe-PLCD3 group. Compared to the blank group, the knock-down group showed higher levels of BAX and P53 expression than the experimental group. Conversely, BCL2 expression was lower in the experimental group than in the blank group. In contrast, the overexpression group exhibited the opposite result (Fig. 6G). According to our database study, PLCD3 plays a key part in the apoptotic process.

### PLCD3 promotes gastric carcinoma via the JAK2/STAT3 pathway

**Fig. 7:**
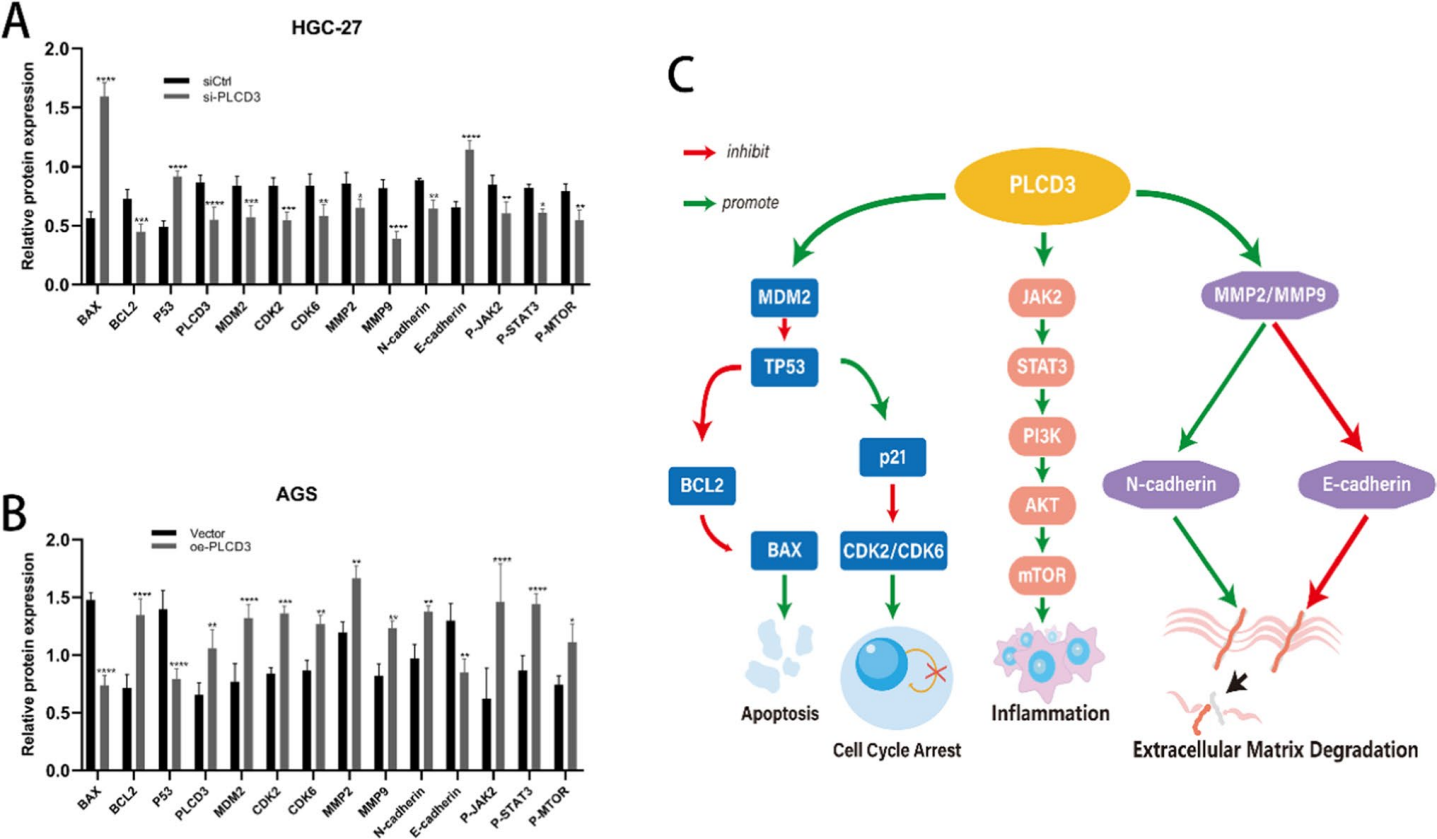
**A** The protein expression results of all PLCD3-related pathways were analyzed in HGC-27 cell lines (*P < 0.05, **P < 0.01, ***P < 0.001, and ****P < 0.0001). **B** The protein expression results of all PLCD3-related pathways were analyzed in AGS cell lines (*P < 0.05, **P < 0.01, ***P < 0.001, and ****P < 0.0001). **C** Working model of the potential function mechanisms of PLCD3 in GC progression

The JAK2/STAT3 signaling pathway is important in tumorigenesis and development [20]. We examined whether PLCD3 promotes gastric carcinogenesis through the JAK2/STAT3 signaling pathway using western blot analysis. According to our findings (Fig. 6H), decreased PLCD3 expression inhibited p-JAK2 and p-STAT3 expression compared to the siCtrl group, while increased PLCD3 expression enhanced p-JAK2 and p-STAT3 expression compared to the Vector group. By contrast, PLCD3 expression had no effect on the protein levels of either total JAK2 or total STAT3. Furthermore, we found that PLCD3 may also be involved in the MTOR pathway. In conclusion, we summarize and analyze the results for several pathways (Fig. 7A–C).

## Discussion

Gastric cancer (GC) is a malignant tumor that originates from the epithelium of the gastric mucosa and is characterized by harmful genetic changes. However, the mechanisms underlying tumour development remain unclear. This study examined the effects of silencing PLCD3 on the proliferation, invasion, and migration of GC cells. Overexpression of PLCD3 had the opposite effect. As a consequence, we used western blot analysis to validate the cycle pathway and the EMT process, which are closely related to proliferation, invasion, and migration. In order to investigate the potential correlation between PLCD3 and apoptosis, as well as the JAK2/STAT3 pathway, we used genomic bioinformatics. Using western blot analysis, we were able to confirm this connection.

PLCD3 is a phospholipase C protein located predominantly in cell membranes [21]. There is the highest level of expression of this gene in the human heart and skeletal muscle, followed by the brain and placenta [22]. PLCD3 is one of three positional candidates in the 17q21 region, where it plays an important role in the phosphoinositide (PI) cycle, which can influence vascular tone and cell proliferation, ultimately leading to atherosclerosis [23]. In previous research, it has been suggested that knocking down PLCD3 may impact neuronal growth processes and may contribute to the proper organization and development of the cerebral cortex and cerebellum by regulating the Ca^2+^ signaling pathways [24]. PLCD3 is also involved in the proliferation and migration of breast tumour cells [25]. However, no studies have been conducted to determine whether PLCD3 plays a functional role in GC.

Our study revealed a significant increase in PLCD3 expression in GC tissues. Furthermore, it was discovered via examination of clinical data from 80 GC patients that PLCD3 is a distinct risk factor for GC OS. Additionally, high PLCD3 levels are correlated with TNM stage and tumor size. In addition, we found that GC cell lines expressed PLCD3 at significantly higher levels than GES1, the typical gastric mucosal epithelial cells. According to these findings, PLCD3 may be involved in the proliferation, invasion, and migration of gastric cancer. A KEGG enrichment analysis was also conducted to determine whether PLCD3 is associated with apoptosis, cell cycle, epithelial-mesenchymal transition, and P53 signaling pathway. PLCD3 was found to significantly inhibit the proliferation and colony-forming abilities of GC cells, supporting this hypothesis. In contrast, the opposite result occurred when PLCD3 was overexpressed.Cell proliferation and the cell cycle are intimately related. A complex interplay between cell cycle proteins and CDKs regulates the intricate process of the cell cycle [26]. PLCD3 knockdown and overexpression affect apoptosis by regulating CDK2 and CDK6 expression, which is consistent with our findings. The wound healing assay and invasion assay provided additional evidence that silencing PLCD3 suppresses the migration and invasion potential of GC cells. The overexpression of PLCD3 in GC cells, however, promotes their migration and invasion capabilities. Metalloproteinases (MMPs) play a crucial role in epithelial-mesenchymal transitions (EMTs) in mesenchymal cells. In addition to being a member of the MMP family, MMP2/9 plays a significant role in the development and progression of various malignancies, particularly ovarian and breast cancers [27]. There is a substantial impact that it has on the occurrence and progression of these cancers. Additionally, E-cadherin and N-cadherin play key roles in epithelial mesenchymal transition (EMT) [28, 29]. We conducted protein immunoblotting experiments in order to provide further evidence of the role that PLCD3 plays in promoting the migration and invasion of gastric cancer. The results of our study demonstrate that PLCD3 facilitates this process by acting on the EMT pathway.

Apoptosis is a form of programmed cell death that plays an important role in maintaining a balancebetween cell death and proliferation [30]. An excessive level of apoptosis may be responsible for degenerative disorders such as Alzheimer's disease and other neurodegenerative diseases, while an insufficient level of apoptosis is a characteristic of many cancer cells [31]. It is therefore important to conduct research on apoptosis in order to improve cancer treatment. JAK2/STAT3 signaling contributes to the development of a tumour inflammatory microenvironment that correlates with the occurrence and development of many human cancers [20]. It is commonly acknowledged that JAK2/STAT3 has a role in the development of gastric cancer [32].

In light of the aforementioned findings, we used KEGG enrichment analysis to investigate PLCD3's function in controlling GC cells’ apoptotic process. We also investigated its connection to the JAK/STAT signaling pathway. According to our results, PLCD3 is enriched in the apoptotic pathway and the JAK/STAT pathway. Western blot assay revealed that PLCD3 knockdown raised BAX and P53 expression and decreased BCL2, while PLCD3 overexpression had the reverse effect. In addition, silencing of PLCD3 inhibited the expression of phosphorylated JAK2 and phosphorylated STAT3, whereas overexpression of PLCD3 enhanced their expression. These results indicate that PLCD3 inhibits apoptosis and promotes the development of gastric cancer through the JAK2/STAT3 signaling pathway.

In summary, we discovered that PLCD3 is a novel therapeutic target for GC. PLCD3 knockdown inhibits proliferation, invasion, and migration of GC cells and leads to apoptosis. PLCD3 overexpression resulted in the opposite outcome. This study provides an important target for future targeted therapy with novel molecules by emphasizing the significant role PLCD3 plays in the development of gastric cancer.

## Data Availability

The datasets generated and analyzed in this study are available upon reasonable request from the corresponding author.
